# Setting the Stage for Self-Regulated Learning Instruction and Metacognition Instruction in Musical Practice

**DOI:** 10.3389/fpsyg.2016.01319

**Published:** 2016-08-31

**Authors:** Hans-Christian Jabusch

**Affiliations:** Institute of Musicians' Medicine, University of Music Carl Maria von Weber DresdenDresden, Germany

**Keywords:** self-regulation, self-regulated learning, metacognition, performance cues, music, practice, memory

Over the last two decades, the concept of self-regulated learning (SRL) has guided scientific approaches to understand successful learning in different fields. Self-regulatory processes were shown to explain achievement differences in students, and SRL may effectively improve achievement in students of different proficiency levels (Zimmerman and Schunk, 2011). Self-regulation refers to “self-generated thoughts, feelings, and actions that are planned and cyclically adapted to the attainment of personal goals” (Zimmerman, 2000, p. 14). Three interacting learning phases have been described: forethought (characterized by goal setting, strategic planning, self-beliefs), performance phase (with the application of strategies, self-observation), and self-reflection (including self-evaluation, attribution, self-reaction). Self-regulated actions and processes include feedback-loops on the personal, behavioral, and environmental level (Zimmerman, 2000). In the educational literature, the terms SRL and metacognition are sometimes used interchangeably (Dinsmore et al., 2008). Although there is a common conceptual core, metacognition and self-regulation are different constructs with different roots. Metacognition was originally referred to as knowledge about and regulation of one's cognitive activities in learning processes (Veenman and van Hout-Wolters, 2006). Central components are metacognitive knowledge, i.e., one's knowledge or beliefs about the interactions between person, task, and strategy characteristics, and metacognitive skills, i.e., the knowledge required for the regulation of and control over one's learning activities (Brown, 1978; Flavell, 1979; Veenman and Spaans, 2005). Metacognition plays a crucial role in successful learning. A large review study based on a meta-analysis of 179 sources identified metacognition as having the strongest influence on school learning (followed by classroom management, quantity of instruction, and social student/teacher interaction) among 30 potential predictor variables (Wang et al., 1990). As a consequence, metacognitive processes are of great interest for pedagogy in various domains.

While there is a wide body of literature on SRL and metacognition in academic disciplines, a limited number of studies have been published on SRL and metacognition in musical practice. Based on findings in advanced music students, S.G. Nielsen proposed a cyclical model which identified the student's problem beliefs, strategy use, self-evaluation, and their interrelations as core elements of successful musical practice (Nielsen, 2001). In this model, the different steps and decisions in the practice process are modulated through metacognitive knowledge and regulation. In their seminal framework for studying SRL in music, McPherson and colleagues applied Zimmerman's principles and dimensions of self-regulation to musical practice and development (McPherson and Renwick, 2001, 2011; McPherson and Zimmerman, 2011; McPherson et al., 2012, 2013). In a longitudinal study, they demonstrated how musical skills developed in 157 children over a 3-year period depending on the degree of SRL, e.g., in the musical subskills sight-reading, playing from memory, and playing by ear (McPherson, 2005; McPherson et al., 2012).

Differences in engaging in self-regulatory processes appear very early in the musical training and account for a large part of a student's subsequent progress (McPherson and Renwick, 2011). The question arises whether specific instruction may facilitate the development of SRL and metacognitive skills especially in young musicians and in early stages of musical education. Implications of observational studies, surveys and case studies led to valuable recommendations for music pedagogy (e.g., McPherson and Zimmerman, 2011; McPherson et al., 2013). In a systematic review, self-regulatory behavior in musicians was strongly related to self-regulation instruction, however, the underlying numbers were small, especially in beginner level musicians (Varela et al., 2014). Intervention studies on SRL- and metacognition instruction are scarce.

In the project reported by Lisboa et al. (2015), the application of a metacognitive strategy was investigated in a young non-expert musician memorizing a piano composition. The authors' scientific focus, the so-called performance cues (PCs) based on recorded thoughts during practice, were previously shown to facilitate memorizing in an expert musician (Chaffin and Imreh, 2002). This benefit was regarded as a result of the musician's increased metacognitive awareness of goals and strategies in memorizing—one crucial element of self-regulated practice (Lisboa et al., 2015). The role of PCs in memorizing music shall briefly be described. Memorizing music and recalling by memory, i.e., learning to play a piece without the score, is one of the challenges in Western music performance tradition (Aiello and Williamon, 2002). How do musicians manage to reliably memorize and recall the enormous amount of information provided by a music score? Two kinds of memory are involved. Serial chaining develops automatically during practice based on implicit, procedural learning and may result in very accurate motor sequences (Rubin, 2006; Chaffin, 2011). Memory lapses, however, may lead to a breakdown of the performance if no “safety net” is available. Less experienced musicians tend to rely on serial chaining, whereas expert musicians additionally establish a mental map of the musical work based on the analysis of the musical structure (Aiello and Williamon, 2002; Chaffin, 2011). Consistent with the skilled memory theory of Chase and Ericsson, expert musicians create a safety net by developing hierarchically ordered retrieval schemes (Chase and Ericsson, 1982; Ericsson and Kintsch, 1995; Aiello and Williamon, 2002; Williamon and Valentine, 2002). They use PCs as landmarks which guide them through the process of recalling and playing the piece (Chaffin and Imreh, 2002; Chaffin et al., 2010; Chaffin, 2011; Ginsborg and Chaffin, 2012; Ginsborg et al., 2012). PCs are established by repeatedly thinking about (a) structural, (b) expressive, (c) interpretative, and (d) basic technical aspects (Chaffin, 2011).

Lisboa et al. (2015) wondered whether music students could benefit from PCs—similarly to expert musicians—and invited an 18-years old piano student with no prior experience in intentionally memorizing music to participate in a single-case study. In order to support the student's efforts to memorize a short piano work the teacher asked her to record her playing-related thoughts on copies of the music score. Concurrent or retrospective reports of thoughts are an established method to gain insight into cognitive and metacognitive processes in musical practice (Nielsen, 1997; McPherson et al., 2013). Specifically, the student was asked to document the features of the music she was paying particular attention to while she practiced and to indicate the respective locations in the score by arrows. The student was not informed about the idea of PCs. In the following lesson, the teacher classified the student's documented thoughts according to the abovementioned aspects (a)–(d). Over the 6½-weeks period of practice, a total of eight reports of the student's thoughts either during practice or while performing from memory were made. 9½ weeks after the end of the practice period the student reconstructed and played the piece again by memory without having played it in the meantime. Video recordings of practice, of performances and of the reconstruction session allowed the researchers to identify the locations at which the student started and stopped playing. The starting points were analyzed in combination with the locations and nature of thoughts during practice and performances. Results showed that (1) thoughts in performances occurred at locations at which thoughts had occurred during precedent practice sessions; (2) thoughts in performances occurred at locations where the student had started playing in precedent practice sessions; (3) locations of thoughts were relatively stable over time but the nature of thoughts at particular locations sometimes changed; (4) reconstructing and playing the piece 9½ weeks later, the student used thoughts from the performance at the end of the practice period as retrieval cues. Altogether, the findings supported the hypothesis that the student's thoughts were PCs.

Lisboa et al. (2015) identified the use of PCs as a metacognitive strategy in a non-expert musician. Although this is a single-case study with no cross-over design, the findings provide important insights into the metacognition and self-regulation of memorizing music by showing that a musician with no history of intentionally memorizing music developed PCs by recording thoughts. The procedure required little specific instruction.

Recommendations on SRL- and metacognition instructions are mainly based on case studies, observational studies and surveys. With their efforts to provide effective practice strategies and address metacognition in the lesson, teachers often do not reach their students (Bathgate and Schunn, 2013; Miksza and Tan, 2015). Most musicians do not feel that their training has assisted them in developing metacognitive skills (Barry and Hallam, 2002). In school learning, effects of SRL instructions were larger when provided by researches instead of regular teachers (Dignath and Büttner, 2008). In a small number of controlled studies undertaken on musical practice, heterogeneous results were seen after short term interventions. Self-evaluation instruction had no significant impact on self-evaluation accuracy or performance in music students after a 5-weeks intervention (Hewitt, 2011). Two weeks of metacognitive instructions by teachers specifically trained in metacognitive teaching resulted in higher performance ratings in music students after 2 weeks of practice when compared with students after control instruction and practicing regularly (Bathgate et al., 2012). Similarly, a positive effect was seen after video-based self-regulation instruction in undergraduate music majors after 5 days of practice (Miksza, 2015). In a non-controlled study, 25 music students developed stronger self-regulatory skills after using a digital tool designed specifically to enhance self-regulation for 12 months (Upitis et al., 2012).

The study by Lisboa et al. (2015) and previous research have opened the door to a fascinating and promising area in music education research. We have the opportunity to continue this line of research and systematically address the questions resulting from their findings, with a special focus on controlled intervention studies and on early stages of musical education. These questions may include: (a) Can the findings of the single-case study by Lisboa et al. (2015) be replicated in a larger series of young musicians and in a controlled study design? (b) Which types and which elements of instruction may support the development of metacognitive skills and SRL in young musicians? (c) To which extent is metacognition and SRL specific (e.g., instrument-, performer-, age-specific; specific to a level of expertise) or general? (d) Does the nature of SRL- and metacognition instruction effective in the individual depend on the variables mentioned in (c) or on personality traits? (e) Are metacognitive skills and SRL acquired in other domains helpful in the musical domain and vice versa? (f) Does (early) instruction of metacognitive skills and SRL in the musical domain have an influence on performance quality in the long-term view or on emotional issues such as performance anxiety or musicians' emotional attachment to their instrument? To further improve our knowledge on the instruction of metacognition and SRL in musical practice will be of great interest to cognitive psychologists and to musicians and music teachers.

## Author contributions

The author confirms being the sole contributor of this work and approved it for publication.

## Funding

HJ is head of the Institute of Musicians' Medicine (IMM) at the University of Music Carl Maria von Weber, Dresden, Germany, and is a full professor paid by the University of Music Carl Maria von Weber. He has received funding from the Dystonia Coalition (NS065701; The Dystonia Coalition is part of the NIH Rare Diseases Clinical Research Network and has support from the NIH Office of Rare Diseases Research and National Institute of Neurological Disorders and Stroke). He has received speaker honoraria from the Berlin University of the Arts, Germany (2014, 2015), from the Hof Symphony Orchestra and the Association of Musicians High Franconia, Germany (2015), from the International Summer Academy Lüneburger Heide, Germany (2015), from the Saxony State Music Council, Germany (2015), from the Klinik Bosse Wittenberg Alexianergemeinschaft GmbH (2015), and from the University of Music and Performing Arts Vienna (2015). The IMM has received free pharmaceutical samples from IPSEN PHARMA GmbH. The IMM has received funding from a private donor without ties to the psychological field.

### Conflict of interest statement

The author declares that the research was conducted in the absence of any commercial or financial relationships that could be construed as a potential conflict of interest.
